# Sickle Cell Disease and Its Respiratory Complications

**DOI:** 10.7759/cureus.28528

**Published:** 2022-08-29

**Authors:** Mashal I Khan, Naomi Patel, Roja T Meda, Surya P Nuguru, Sriker Rachakonda, Shravani Sripathi

**Affiliations:** 1 Internal Medicine, Khyber Girls Medical College, Peshawar, PAK; 2 Research, Smt. Nathiba Hargovandas Lakhmichand (NHL) Municipal Medical College, Ahmedabad, IND; 3 Medicine, Narayana Medical College, Nellore, IND; 4 Internal Medicine, Kamineni Academy of Medical Sciences and Research Center, Hyderabad, IND; 5 Medicine, Bogomolets National Medical University, Kyiv, UKR; 6 Surgery, Bhaskar Medical College, Hyderabad, IND

**Keywords:** pulmonary screening, respiratory, pulmonary, asthma, pulmonary hypertension, venous thromboembolism (vte), acute chest syndrome (acs), sickle cell disease complications, sickle cell disease: scd

## Abstract

Sickle cell disease (SCD) is a hematological disorder that is inherited in an autosomal recessive (AR) fashion. It is caused by mutations in the genes encoding for the globin apoprotein of hemoglobin (Hb), leading to diminished oxygen-carrying ability. Its pathophysiologic mechanism affects multiple organ systems, making it crucial to understand the complications of SCD and find the best ways to prevent and treat them. Some important ways that SCD manifests in the respiratory system are acute chest syndrome (ACS), pulmonary hypertension (PH), asthma, and venous thromboembolism (VTE). This article summarizes their salient features, including pathogenesis related to the adverse outcomes, screening practices, and management guidelines, with the intent to provide greater insight into forming better practices that increase the quality of life in SCD patients.

## Introduction and background

Sickle cell disease (SCD), the first inherited disease to be described on the molecular level in 1949 by Pauling et al., is one of the most common inherited hemolytic anemias worldwide, affecting around 250,000 births per year [1,2]. It is estimated that SCD affects roughly 100,000 Americans, the majority of whom are African American, with the second most commonly affected population being Hispanic [3]. It is a group of diseases that occur due to a point mutation in the β-globin chain of hemoglobin (Hb), switching out glutamate for valine at codon 6 of the β-hemoglobin allele [1,3]. This leads to the Hb molecule being unable to withstand the same amount of oxidative stress that a normal molecule of Hb would, causing the red blood cell (RBC) to sickle, taking the shape of a crescent during stressful conditions. This sickling of RBCs can lead to hemolytic anemia, vasculopathy, and occlusion of the blood vessels, resulting in acute and chronic organ damage [4]. The prevalence of sickle cell trait (HbAS) and SCD (HbSS) is higher in African populations due to the protection and better outcomes the heterozygously inherited sickle cell trait provides against endemic malaria on the continent [5]. SCD is inherited in an AR pattern (HbSS), meaning both parents must possess the allele for a child to inherit it homozygously, manifesting the disease form [6].

Sickle cell anemia (SCA) differs from SCD in that it is asymptomatic and benign in the majority of cases due to low levels of abnormal hemoglobin S (HbS) and can very rarely undergo sickling in unusual or low oxygen environments such as the renal medulla, where it causes painless hematuria [7]. SCD, on the other hand, has much graver consequences if it goes undiagnosed. For this reason, the National Institutes of Health (NIH) recommended as early as 1987 that all newborns be screened for SCD in the US. However, this recommendation was only implemented in all 50 states in 2006 [8]. Newborn screening is imperative because SCD is asymptomatic at birth due to the persistence of fetal hemoglobin (HbF), which contains gamma chains instead of beta chains, leaving it unaffected by the sickle cell mutation. As HbF levels fall by eight to ten weeks of life, the SCD crisis may begin. Manifestations can include splenic sequestration crisis, dactylitis, and respiratory complications such as acute chest syndrome (ACS), pulmonary hypertension (PH), asthma, and pulmonary thromboembolism [7,9]. It is essential to understand the effects of SCD on the respiratory system because the most common cause of death in these patients is a result of respiratory complications, mainly ACS and PH [9].

Hb electrophoresis confirms the diagnosis of SCD, a process in which the Hb molecules are separated by their size and charge, followed by monitoring their migration pattern towards negative or positively charged electrodes to differentiate the subtype of Hb present [2]. SCD is managed in various ways, beginning with counseling patients regarding the triggers of crises, screening programs to assess respiratory function, and finally, preventing severe infections with encapsulated organisms such as pneumococcus, to which they are more predisposed due to functional asplenia, with the help of vaccines and penicillin injections. Also, pharmacological therapy with hydroxyurea increases the concentration of HbF, thereby preventing the sickling of Hb [5,10]. This article aims to provide an overview of how the pathophysiology of SCD complicates into ACS, PH, asthma, and pulmonary thromboembolism, as well as their important screening practices and respective management.

## Review

Pathophysiology of SCD

SCD is a form of hemoglobinopathy in which gene mutations in the globin apoproteins alter the expression of Hb [4]. As oxygen binds to iron, which is typically carried by globin, an abnormality of this component disrupts the normal Hb-oxygen interaction. HbS polymerizes in low-oxygen environments, so entrance into the microcirculation progressively lowers oxygen levels, causing HbS to enter a deoxygenated state and polymerize after reaching the pulmonary circulation [4,11]. The repetitive process of polymerizing and depolymerizing results in irreparable damage to the RBC cytoskeleton and alteration of the RBC ensues, evidenced by sickling on the peripheral blood smear [11]. These damaged sickled RBCs accumulate, causing the symptomatology of SCD, including vaso-occlusion and hemolytic complications [4]. The concentration of HbS determines the tendency toward forming polymers [4]. Sickle erythrocytes are predictably heterogeneous due to their diverse patterns of membrane injury [12]. In addition, the level of HbF in each erythrocyte impacts its resilience toward stress and hence, the ability to survive the circulatory dynamics [12].

Acute chest syndrome

ACS is defined as the appearance of new pulmonary infiltrates on a chest X-ray that corresponds with alveolar consolidation, along with symptoms such as chest pain, fever, tachycardia, cough, wheezing, and hypoxemia [13]. It is the second most common cause of hospitalization in patients with SCD, preceded only by vaso-occlusive crisis [9]. The outcome is highly variable and can range from self-resolution to acute respiratory failure and even death [14]. The pathogenic cause of ACS has not been narrowed down to one inciting point; instead, three significant factors have been studied as the triggering mechanisms. These include infectious causes such as pneumonia or other systemic infection, fat embolism, and pulmonary infarction due to vaso occlusion by sickled RBCs [15,16]. A multicentre study was performed by the National Acute Chest Syndrome Study Group, documenting 671 cases of ACS in 538 patients. An infectious agent was identified in 54% of these cases [16]. It has been suggested that people suffering from SCD incur an immoderately high inflammatory response to pulmonary pathogens [17]. A 2003 study was conducted by Holtzclaw et al. to test this hypothesis. Both transgenic sickle mice and control mice were injected with lipopolysaccharide (LPS) endotoxin [17]. It was noted that at baseline, the sickle mice had increased levels of leukocytes and soluble vascular cell adhesion molecule 1 (sVCAM-1) in their circulation [17]. A significantly enhanced response to the LPS challenge was seen in the sickle mice compared to the control mice, denoting a baseline "proinflammatory'' state, which suggests it to be the cause of increased levels of lung injury after exposure to lower levels of endotoxins. This inflammatory state explains the higher predisposition to lung injury in the presence of infection [17].

Another major cause of ACS is fat emboli syndrome. It follows a vaso-occlusive crisis in bones, such as the pelvis and femur, leading to infarction, edema, and ultimately necrosis of the bone marrow, subsequently releasing fat molecules into the bloodstream [15]. This fat travels to the lungs, causing a pulmonary fat embolism, which promotes secondary inflammation and hypoxemia, eventually leading to constriction of pulmonary blood vessels and hence PH [18]. PH worsens the disease progression in ACS. A 2008 study performed by Dessap et al. shows that the mortality rate in ACS has a higher association with PH, specifically with tricuspid jet velocity values of 3 m/second or more [19]. Fat emboli syndrome is diagnosed by the presence of alveolar macrophages stained positively for oil red O, which stains lipids [20]. With the help of bronchoscopy, the National ACS Study Group diagnosed fat emboli syndrome based on lipids containing alveolar macrophages in 16% of the studied ACS patients [16].

The third factor implicated in the development of ACS is the occlusion of pulmonary blood vessels by sickled RBCs, leading to infarction. A 2011 study used computed tomography-pulmonary angiography (CT-PA) to investigate the presence of thromboembolism in ACS patients, with a 17% prevalence found [21]. Some risk factors predispose to the development of ACS in the setting of a vaso-occlusive crisis. One implication is the higher levels of sickled Hb in the body, which results in increased viscosity, predisposing to vaso-occlusive crisis and, therefore, the development of ACS [15,16]. It has also been shown that the Hb count drops suddenly at the onset of ACS due to hemolysis. This is suggested by the increase in lactate dehydrogenase (LDH) level, which is released due to the destruction of RBCs [15,16]. This acute hemolysis may be the cause of lung injury [15,16]. Another feature noticed in SCD is that at baseline, patients with SCD generally have an increased platelet count due to functional asplenia [15,16]. This platelet count has been seen to drop before and during ACS events. A drop below 200 × 103 /µl is implicated in both the development of multilobar ACS as well as the need for mechanical ventilation [15,16]. No specific screening guideline has been proven effective in screening for ACS prior to its occurrence [8]. It is recommended to take a thorough respiratory history and examination from all asymptomatic patients with SCD and follow up on any pertinent findings [8]. In addition, every patient that develops lower respiratory tract symptoms, regardless of fever, should be evaluated for the presence of ACS by measuring oxygen saturation and performing a chest X-ray [8,10]. Management of patients with ACS includes hospitalization and monitoring oxygen saturation, which is to be kept above 95% with the help of supplemental oxygen [8,10]. Administration of antibiotics is strongly recommended and should include intravenous cephalosporin and oral macrolide [8,10]. The patient should also be monitored for complications such as bronchospasm, hypoxemia, and acute anemia. In the case of rapid deterioration or progression of ACS, which is demonstrated by the inability to maintain oxygen saturation over 90% despite supplemental oxygen therapy, respiratory distress, increasing pulmonary infiltrates on imaging, or a drop in Hb despite receiving a simple transfusion; further measures should be taken [8]. These include consulting with specialists in hematology, critical care, and apheresis to perform an exchange transfusion on the patient [8]. Another important preventive therapy is the use of incentive spirometry [8,10]. A study by Bellet et al. shows that implementing an incentive spirometry regimen significantly reduced the development of ACS in SCD patients hospitalized for chest or back pain [22]. Patients should also be offered pain control in the form of opioids or non-steroidal anti-inflammatory drugs (NSAIDs) during acute episodes to help maintain adequate respiratory ventilation and prevent further complications [23].

Asthma

Asthma is a common disease associated with SCD and ACS due to its high prevalence in the general population. It affects 12% of all children in the US, around 15% to 20% of African American children, and about 9% of African American adults [24,25]. Similar to non-SCD patients, asthma attacks in SCD are triggered by upper respiratory infections, cold weather, cigarette smoke, allergies, and pets [25]. It presents with recurrent episodes of wheezing, intercostal and supraclavicular retractions, shortness of breath, a cough that is worse at night and exercise intolerance, among other findings. These symptoms are caused by bronchial hyperresponsiveness, episodic bronchoconstriction, and acute-on-chronic inflammation [25]. The exacerbations give rise to mucous plugging, ventilation-perfusion mismatch, and hypoxemia [26]. This hypoxemia causes sickling of the RBCs in the blood vessels, predisposing them to ACS [25]. Although there is a shortage of research investigating the reason asthma and SCD occur together, it has been shown to have a clear association with increased cases of ACS, cerebrovascular accidents, and PH, warranting the question of its connection with SCD [24-26]. A 2006 prospective study was performed on a cohort of 291 African American infants under six months of age with HbSS that were followed beyond the age of five years [24]. Among this cohort, the study compared the incidence of ACS between those diagnosed with and without asthma. Asthmatic patients suffered twice the number of ACS episodes compared to those without [24]. Pediatric patients with asthma get their first episode of ACS earlier than non-asthmatic patients, with the median age being 2.4 years versus 4.6 years for the latter [24]. Asthma is also associated with increased mortality in SCD patients [27]. This is supported by a 2007 study conducted by Boyd et al. on 1,963 SCD patients followed for 18,495 patient years. It demonstrates that asthmatic SCD patients have an all-cause mortality risk that is two times greater than non-asthmatic patients [27]. Also, it showed that the median lifespan for patients with and without asthma was 52.5 and 64.3 years, respectively [27]. The diagnosis of asthma is made clinically, but pulmonary function tests (PFTs) and a methacholine challenge test can also be carried out to aid in diagnosis [28]. PFTs are normal at baseline, but a reversible obstructive pattern may be present during exacerbations [28]. Due to an inadequate number of studies done on asthma with SCD, there is insufficient knowledge regarding traditional asthma therapies. They are treated similarly to non-SCD asthma patients [25]. Corticosteroids are given to patients with asthma exacerbations. The benefits of administering corticosteroids outweigh the concern of precipitating a potential vaso-occlusive crisis. Additional options for management include supplemental oxygen therapy, inhaled short-acting β2 agonists, and intravenous magnesium sulfate for severe cases [25].

Pulmonary hypertension

**Figure 1:**
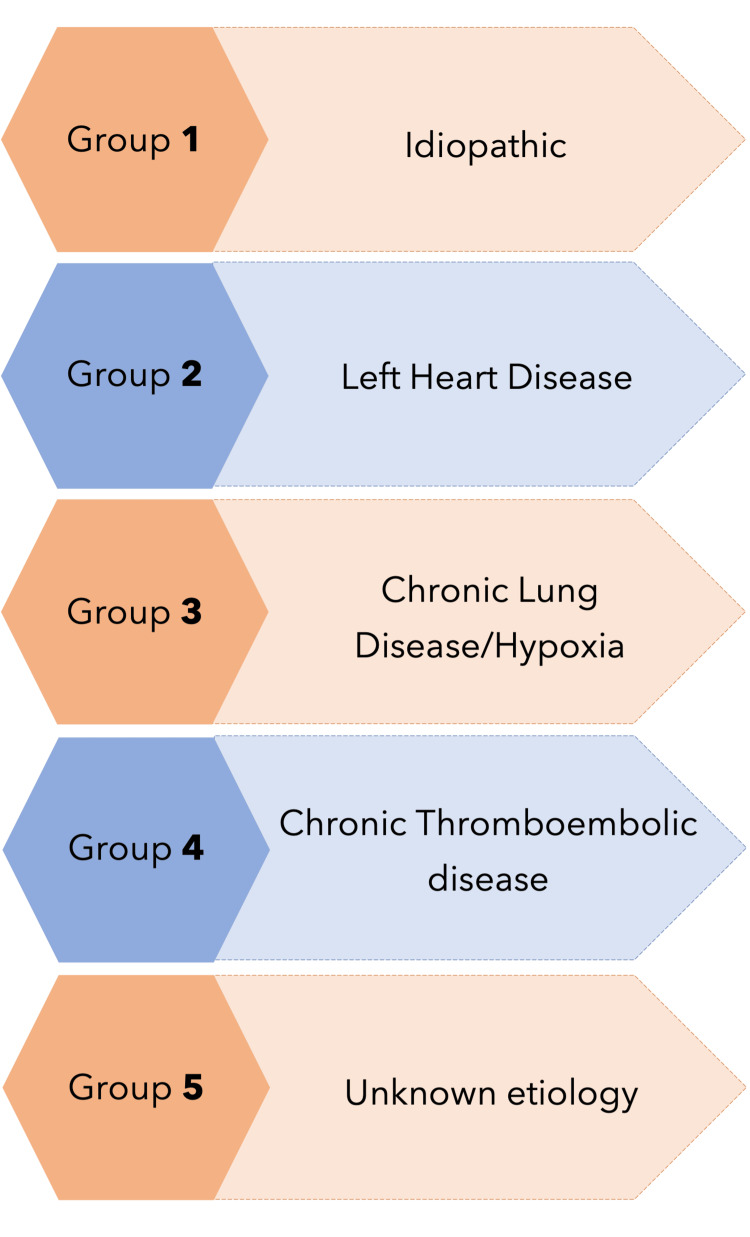
Classification of Pulmonary Hypertension According to Cause

PH is defined as a mean pulmonary arterial pressure of ≥25 mmHg at rest and is measured with the help of right heart catheterization [29,30]. SCD affects the vascular structure, leading to PH as a complication, which affects anywhere from 6% to 10% of adults with SCD [29,30]. PH has been classified into five groups, as shown in Figure 1.

According to this guideline, SCD caused by PH is categorized into five groups due to patients having features of both precapillary and postcapillary PH; having a lower pulmonary vascular resistance (PVR) compared to group 1, and the coexisting presence of thromboses in some patients that is similar to group 4 [1,31]. The symptoms of PH are nonspecific and can present as shortness of breath or dyspnea on exertion, fatigue, chest pain, and syncope. Signs of right heart failure, such as edema and jugular venous distension, can also be present on examination but usually appear in the late stage of the disease [32]. There are two factors causing hemolysis in SCD. The first factor is intracellularly polymerized HbS mechanically shearing the RBC membranes along with an overall increase in oxidative and metabolic stress. This causes reduced fluidity of the RBC membrane, promoting hemolysis. The second factor is related to the increased expression of phosphatidylserine residues, which predisposes them to destruction [33]. In comparison to people with normal red cells, there are increased levels of hemolysis, which releases factors predisposing to imbalanced vascular dynamics [33]. One of the critical issues in PH is the disparity between vasoconstrictors and vasodilators [33]. A study proved that the destruction of RBCs releases arginase. This enzyme breaks down arginine, a key substrate of the enzyme nitric oxide synthase (NOS), during the formation of nitric oxide (NO), a potent vasodilator. The lack of arginine acts as an uncoupler to the activity of NOS, causing the formation of reactive oxygen species instead of NO [34]. Another molecule released by the destroyed RBCs is adenosine diphosphate (ADP). It is an important stimulator in the coagulation pathway leading to clot formation [35]. Chronic vasoconstriction, chronic pulmonary thromboembolism, along with increased hypoxic response due to anemia, low oxygen saturation, and impeded micro-vasculature are all pre-capillary causes of PH [36]. Post-capillary PH is caused by left ventricular dysfunction and is attributed to the relative systemic hypertension caused by the chronic anemic state [36]. Due to the increased systemic vascular resistance, the left heart has a higher afterload to work against, leading to ventricular dilation and concentric hypertrophy. The subsequent backup of pressure causes post-capillary pulmonary hypertension [36].

Echocardiography is used as a tool to assess for PH by looking for findings relating to increased pulmonary vascular pressures such as enlargement of the right atrium, hypertrophy or dilation of the right ventricle, septal shifting, and inferior vena caval changes such as increased diameter with decreased inspiratory collapse, and also by combining these with measurement of tricuspid regurgitation velocity (TRV) [37]. Guidelines provided by the American Thoracic Society suggest that SCD patients should be screened once for PH between the ages of 8 and 18, with the help of Doppler echocardiography [36,38]. Echocardiography is performed every one to three years or based on findings associated with increased risk such as exertional dyspnea, decreased exercise capability, thromboembolic history, resting oxygen saturation of less than 96%, examination findings suggesting right heart overload, increased TRV of ≥2.5 m/sec, and pertinent laboratory values [36,38]. For further management, patients with findings suggestive of PH are referred to specialized PH treatment centers that are experienced with SCD patients [37]. The definitive diagnosis remains determined by the results of a right heart catheterization [30]. The therapeutic measures to combat the development of complications of PH in SCD patients include the augmentation of SCD therapy with the goal of reducing hemolytic anemia [1]. The first-line treatment that has shown benefits is hydroxyurea [39]. It increases the concentration of HbF, decreases the number of vaso-occlusive events and ACS, and has shown an overall improvement in the survival of HbSS patients [38]. If hydroxyurea is not well tolerated, chronic RBC transfusion therapy can be administered [39]. It improves the delivery of oxygen to tissues by reducing the formation of sickle RBCs and the rate of hemolysis [40]. There are risks of iron overload and alloimmunization, but the benefits of its use should be assessed individually [38]. Pharmacological therapies targeting PH have also proven effective. In 2005, a study performed by Machado et al. evaluating the efficacy of Sildenafil therapy in 12 patients with SCD and PH, concluded it to be safe and beneficial by improving exercise tolerance as well as vascular pressure in these patients [41]. Another study showed that SCD patients have a higher level of endothelin-1 vasoconstrictor; thus, using endothelin antagonists such as Bosentan has promising results [42].

Venous thromboembolism

SCD is a state of disrupted hemostatic balance, with the scale tipped towards hypercoagulability, making thromboembolism prevalent in the SCD population and hence, a significant cause of morbidity and mortality [43]. A large study using the National Hospital Discharge Survey investigated 1,804,000 SCD admissions from 1979 to 2003, showing that the prevalence of pulmonary embolism (PE) in hospitalized SCD patients less than 40 years of age was approximately 3.5 times higher than in African-American controls [43]. Another study demonstrated the incidence of SCD-PE to be increased by almost 50-100-fold in hospitalized patients compared to non-SCD patients [44]. These studies prove that SCD is a hypercoagulable state with a higher predisposition towards thromboembolic complications than the general population. The pathogenesis behind the development of thromboembolic complications is based on Virchow's triad, consisting of a hypercoagulable state, endothelial dysfunction, and hemostasis [45]. There are a variety of ways that this state of imbalance can be exacerbated. One mechanism of vaso-occlusion is the expression of procoagulant phosphatidylserine on the membranes of sickled RBCs during vaso-occlusive episodes [45]. These RBCs adhere to the endothelium, attracting more red cells, white blood cells, and platelets, causing occlusion [45,46]. The endothelial cells also activate proinflammatory molecules, which drive this process further by inducing the expression of more surface adhesion molecules [45,47]. There is also evidence pointing towards a decrease in the levels of anticoagulants such as protein C and protein S in the body, perpetuating a prothrombotic state in SCD [45,47]. Another mechanism by which a thrombotic state is propagated is through platelet activation and aggregation [48]. This accelerated platelet activation occurs due to ruptured RBCs releasing free Hb, which interacts with NO to induce vasoconstriction and thus platelets become activated [48]. Some factors that provide evidence for continuous platelet activation are the increased expression of P-selectin on platelets, plasma soluble factors 3 and 4, platelet-derived soluble CD40 ligand, and β-thromboglobulin [48].

The suspicion of venous thromboembolism (VTE) warrants the need for lab investigations. Generally, a D-dimer test is used to aid in diagnosing VTE due to its high negative predictive value [49]. However, the pathophysiology of SCD renders the D-dimer test unreliable due to the continuously activated coagulation cascade [49]. For suspected cases of upper or lower extremity DVT, the use of compression ultrasound is beneficial [49]. Another investigation used to diagnose PE is computerized tomographic pulmonary angiography (CTPA). It typically has high specificity, but in SCD patients, the findings may be inaccurate [21]. This is because the filling defects visualized on CTPA may represent the increased prevalence of in situ pulmonary thrombosis in ACS patients and the in situ sickling present in the absence of ACS [21]. Radionuclide scans (ventilation/perfusion scans) are argued to be better due to their advantages, which include reduced radiation exposure, well-defined diagnostic criteria, and no concern of contrast-induced kidney injury in SCD patients who are already predisposed to organ dysfunction [49]. There is no established guideline regarding the management of VTE in SCD patients [50]. Currently, some research has shown that anticoagulant drugs such as warfarin, acenocoumarol, and heparin are beneficial in preventing vaso-occlusive crises in SCD [50]. There are also no specific guidelines outlining the prophylaxis for VTE in SCD, so the decision to prescribe prophylactic drugs should be tailored to individual patient history and comorbidities, along with the risk for bleeding [51].

Limitations 

SCD has a complex pathology with many complications, only a few of which have been described here, limited to the respiratory system. The respiratory complications of SCD are not limited to those discussed in this article. The complex pathophysiology of SCD can have a multitude of downstream effects. The complications are not limited to their association with SCD and have many predisposing and causative factors aside from SCD. This article has only discussed some of their correlating factors and the mechanism by which SCD would cause them. The resources utilized to gather data for this article were limited to PubMed.

## Conclusions

SCD is an inherited hematological disorder of Hb that causes RBCs to polymerize in hypoxic conditions, subsequently affecting all organ systems. As demonstrated in this review, SCD has important manifestations in the respiratory system, including a strong association with asthma and complications such as ACS, PH, and pulmonary VTE. It highlights the clinical significance of screening for and identifying these complications in conjunction with prevention and treatment, which has proven effective in preventing severe disease and death in SCD patients. Although there are no specific screening guidelines for ACS, all asymptomatic patients with SCD should undergo a thorough respiratory history and examination with follow-up on any concerning findings. Also, every patient who develops lower respiratory tract symptoms, regardless of fever, should be evaluated for the presence of ACS. A higher number of SCD patients have concurrent asthma than the general population. An attack triggers earlier and higher numbers of ACS episodes, along with more significant mortality, creating a greater need for a timely diagnosis of potential asthma. All SCD patients should be scanned for PH regularly and with greater frequency depending on the presence of risk-associated symptoms. SCD patients should also be screened for PE, a respiratory complication caused by VTE. Appropriate management of these complications creates a positive outlook for many patients. Many current practices overlap with or comprise the guidelines already established for respiratory disease in the general population. This article brings attention to the importance of further exploration in establishing universal SCD screening practices and management guidelines to reduce the rate of hospitalizations and mortality, along with improving the quality of life in these patients. While some complications have been extensively researched, others warrant additional investigations into the pathophysiologic mechanisms of SCD. A great extent of analysis is still required in the timely recognition of SCD complications, along with the formation of clinical screening guidelines and management protocols with proven mortality benefits specific to SCD patients.
